# Immune response associated with Toll-like receptor 4 signaling pathway leads to steroid-induced femoral head osteonecrosis

**DOI:** 10.1186/1471-2474-15-18

**Published:** 2014-01-15

**Authors:** Lei Tian, Qi Wen, Xiaoqian Dang, Wulin You, Lihong Fan, Kunzheng Wang

**Affiliations:** 1 Department of Orthopedics Surgery, The Second Affiliated Hospital, College of Medicine, Xi’an Jiaotong University Xi’an, Xi’an, Shaanxi 710061, PR China; 2 Department of Orthopedics Surgery, Shandong Provincial Hospital Affiliated to Shandong University, Jinan, Shandong 250021, PR China; 3 Department of Orthopedics Surgery, the Second People’s Hospital of Shaanxi Province, Xi’an, Shaanxi 710061, PR China

**Keywords:** Osteonecrosis, Femoral head, Toll-like receptor 4, Corticosteroids, Rat

## Abstract

**Background:**

Femoral head osteonecrosis is frequently observed in patients treated with excessive corticosteroids. The objective of the current study was to establish a rat model to investigate the disruption of immune response in steroid-induced femoral head osteonecrosis via Toll-like receptor 4 (TLR4) signaling pathway.

**Methods:**

Male SD rats were divided into the treatment group (group A) and the model group (group B) consisting of 24 rats each, and were injected intramuscularly with 20 mg/kg methylprednisolone (MP) for 8 weeks, once a week. The rats in group A were injected intravenously with 7.5 mg/kg TAK242 before each MP administration. A control group (group N) consisted of 12 rats were received saline injection. All animals were sacrificed 8, 10 and 12 weeks from the first MP injection, respectively. Histopathological analysis was performed and the concentration of tartrate-resistant acid phosphatase (TRAP) in serum was tested. The signaling molecules including TLR4, MyD88, NF-κB p65 and MCP-1 were detected by immunohistochemistry, quantitative real-time PCR and Western blot.

**Results:**

Femoral head osteonecrosis was observed in the model rats, and the concentration of TRAP and positive staining of all signaling molecules increased significantly in group B compared with that in group A and group N. Compare with the control group, the mRNA expressions and protein levels of all signaling molecules were enhanced significantly in group B, but no significant in group A.

**Conclusions:**

Corticosteroids can induce femoral head osteonecrosis by disturbing the immune response via TLR4 signaling pathway. These findings suggest that the disruption of immune response play a role in the pathogenesis of osteonecrosis.

## Background

Steroid-induced osteonecrosis of femoral head often leads to progressive collapse and followed by degenerative arthritis. It is frequently caused by excessive corticosteroids administration. The adverse effects of corticosteroids such as obesity, hyperlipidemia, osteoporosis and Cushing syndrome have limited their clinical use, particularly at high dose and for prolonged administration [1,2]. Recently, corticosteroid was considered a primary risk factor in non-traumatic osteonecrosis. However, the underlying pathogenesis of steroid-induced femoral head osteonecrosis remains unclear [3-6], and effective prophylactic treatment has not yet been identified. Recent studies have implicated that the pathogenesis may be multifactorial, which includes, osteoporosis, intravascular fat emboli, vascular coagulation and abnormal gene expression, maybe all of which interact and finally lead to the lesion [7-10].

To examine the pathogenesis of steroid-induced osteonecrosis, a disseminated intravascular coagulation shock rabbit model has been developed in which osteonecrosis is induced by administering lipopolysaccharide (LPS) and methylprednisolone (MP) [11]. However, this model shows metaphysic osteonecrosis and not the epiphysic necrosis that is observed in human patients. To overcome these limitations, a rat model has been developed in which osteonecrosis is induced by excessive MP [12-14]. The rats genome is 90 percent similar to the human genome, and thus the pathological characteristics of osteonecrosis in rats and human may be similar. In addition, the femoral anteversion and neck-shaft angle of rats are similar to that of humans, and the mechanical property of the structures can be similar.

**Figure 1:**
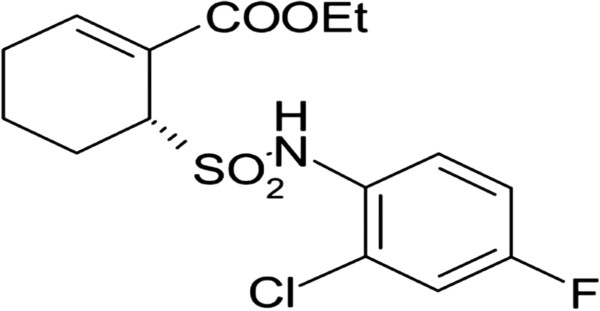
Structure of TLR antagonist: TAK-242 (Resatorvid).

Toll-like receptor 4 (TLR4) is one of the most common members in Toll-like receptors (TLRs) family, and it is also the most thoroughly studied. As a pattern recognition receptor, TLR4 has close relationship to autoimmune or inflammatory diseases [15,16]. TLRs antagonists currently under development are structural analogs of agonists and probably bind to the receptor but fail to signal. TAK-242, [ethyl (6R)-6-[N-(2-chloro-4-fluorophenyl) sulfamoyl] cyclohex-1-ene-1-carboxylate] (Figure 1), is a TLR4 antagonist that is in a Phase III trial for the treatment of inflammatory and autoimmune diseases. TAK242 acts as a selective inhibitor of signaling from the intracellular domain of TLR4 [17,18], also inhibits activation of nuclear factor-κB (NF-κB) and ligand-independent NF-κB activation resulting from over-expression of TLR4. MyD88 plays an important role in the MyD88-dependent TLR4 signaling pathway, and can mediate the signals transduct to the downstream. NF-κB, one of the most important transcriptional signaling molecules, participates in the downstream inflammatory pathway and TLR4 signaling pathway, and its essential role in osteoclastogenesis has been demonstrated genetically. NF-κB can transduce signals by recruiting adaptor molecules such as myeloid differentiation primary response gene 88 (MyD88) [19]. In addition, NF-κB can activate monocyte chemotactic protein-1 (MCP-1) that induces the proliferation of monocytes/macrophages, which finally form osteoclasts.

Accordingly, the objectives of this study were to clarify the pathogenesis of steroid-induced femoral head osteonecrosis and investigate the role of immune response disorder in osteonecrosis via TLR4 signaling pathway using a rat model of steroid-induced femoral head osteonecrosis.

## Methods

### Animals

Male SD rats weighing 230-250 g (8-12 weeks old) were obtained from the Animal Center of the Medical College of Xi’an Jiaotong University (Xi’an, China). They were specific pathogen free animals (SPFA) housed in clean, temperature- and humidity-controlled SPFA chamber with a room temperature of 22 ± 2°C, a relative humidity of 50 ± 5%, unlimited food and water, and a 12 h light/dark cycle.

### Experimental protocols

All experiments abided by the guidelines of the Ministry of Health, Culture, Science and Technology of China. The experimental protocol was approved by the Animal Ethics Committee of Xi’an Jiaotong University (Xi’an, China). Forty-eight animals divided into the treatment group (group A) and the model group (group B) consisting of 24 rats each, all were injected intramuscularly with 20 mg/kg MP (Pfizer Manufacturing, Belgium NV) via bilateral gluteus maximus alternately for 8 weeks, once a week. Based on a logistic regression model, laboratory variables before steroid injection were assessed to determine whether they demonstrated any association with the risk of ON. The rats in group A were injected intravenously with 7.5 mg/kg TAK242 (Haoyuan Chemexpress Co., Ltd., Shanghai, China) before each MP administration, also for 8 weeks, once a week. On the basis of TAK242 dosage to human pharmacy clinical trials (Phase III), and the dose conversion formula between human and rat, the final dosage was calculated. The control group (group N) consisted of 12 rats that were fed and housed under identical conditions but were received saline injection.

The rats in all groups were sacrificed by overdose of anesthesia respectively at weeks 8, 10 and 12 from the first MP injection, and the femoral heads were harvested. The left femoral heads of all rats were preserved in -70°C cryogenic freezer immediately after sacrifice, half of which were performed RNA isolation and the others were performed protein isolation, respectively. The right one was collected and furthered evenly divided into two subgroups: one was fixed with 10% formalin (0.1 M phosphate buffer, pH 7.4), and the other was fixed with 2.5% glutaraldehyde solution in 4°C. Blood was collected from the inferior vena cava into the anticoagulant tubes at the time of sacrifice and was centrifuged immediately. The supernatant serum was stored in -70°C cryogenic freezer for Enzyme-Linked Immunosorbent Assay (ELISA) analysis. The controlled animals were managed in the same way at weeks 8, 10 and 12, respectively.

### ELISA analysis

TRAP concentrations were determined using commercial ELISA kits (R&D Systems, USA). Briefly, 50 μL of standards, supernatant samples and controls were added to each well of the coated microplate. Then, 50 μL specific biotin-conjugate antibodies were added to each well, followed by 2 h incubation at 37°C. The liquid of each well was discarded and the samples were incubated for another 2 h at 20-22°C. The wells were then washed three times, and 100 μL Streptavidin-HRP was added. After further incubation for 1 h, the wells were washed again and 100 μL substrate chromogen solutions were added. After another incubation of 1 h and washing, 90 μL substrate solutions were added, followed by incubation at room temperature for 30 min. Finally, 50 μL of stop solution was added. The absorbance of each well was read within 30 min, spectrophotometrically at λ 450 nm. The mean O.D. value for each standard and sample was calculated accordingly. All values were subtracted by the mean value of the zero standard before result interpretation. Finally, the curve equation of TRAP concentrations was calculated, values were expressed as pg/ml protein, and the standard curve was plotted according to the amount in all the samples using GraphPad Prism 5.0 software (GraphPad Software, Inc., San Diego, California, USA).

### Histopathological staining

The bilateral femoral heads harvested from the animals were immediately fixed with 10% formalin (0.1 M phosphate buffer, pH 7.4) at 4°C for 24 h, and then washed with 0.2 M phosphate buffer (pH 7.4). Then, the specimens were decalcified with 10% EDTA neutralized with sodium sulphate buffer for about 4 weeks. After decalcification, the tissues were embedded in paraffin and cut in the coronal plane into 4 μm thick sections with a microtome. Some of the sections were processed for routine hematoxylin-eosin staining to assess the general architecture and injury of the tissue. The stained sections were viewed at a magnification of 200 times and photographed with eclipse 50i optical microscope imaging system (Nikon, Co. Ltd, Toyko, Japan), and the images were analyzed by Image-Pro-plus sofeware (Media Cybernetics, Baltimore, MD).

### Immunohistochemistry

The rest femoral head tissue sections obtained in the above process were processed immunohistochemically to detect the presence of TLR4, MyD88, NF-κB p65 and MCP-1 using the avidin-biotin-peroxidase complex (ABC) method. After permeabilized in phosphate-buffered saline (PBS) with 0.3% Triton-X 100 (pH 7.4) for 30 min and then 0.3% H_2_O_2_ for 1 h in order to block endogenous peroxidase, the sections were incubated with primary mouse anti-rat monoclonal antibodies (rabbit anti-rat, 1:200, Santa Cruz Biotechnology, USA) against TLR4, MyD88, NF-κB p65 and MCP-1 separately in a solution consisting of 1% bovine serum albumin and 0.05% sodium azide in 0.1 M PBS for 24 h at 4°C. After three washings in PBS, the specimens were exposed to biotinylated goat anti-mouse IgG diluted 1:200 in PBS for 4 h at room temperature. The peroxidase reaction was then developed for 10 min in 0.05 M Tris buffer (pH 7.6) containing 0.02% 3, 3-diaminobenzidine tetrahydrochloride and 0.006% H_2_O_2_. The sections were photographed using the eclipse 50i optical microscope imaging system, and the images were analyzed by Image-Pro-plus sofeware.

Positive staining for the signaling molecules in subchondral area of the femoral heads would be visible as brown puncta and bundles distributed in the bone marrow, periosteum and bony trabeculae. The positive brown staining provided in the protocol for each antibody was used as positive control. Samples without primary antibodies were used as negative controls. The intensities of immunostaining for TLR4, MyD88, NF-κB p65 and MCP-1 from group A, B and N were quantitatively analyzed. It is important that all the sections were analyzed at the same time for the quantitative study. The images obtained were analyzed at a magnification of 200 times by quantitative integrated optical density. The area of each analyzed tissue was approximately the same. We evaluated the integrated optical density, which referred to the sum of all the pixel intensity or density values in a given region [20]. Ten different regions in each tissue were randomly selected and the numerical values obtained from the ten regions were averaged to represent the specified marker in a given tissue.

### Quantitative real-time PCR (RT-qPCR) analysis

Total RNA was isolated with RNase-free DNase from the femoral head by using TRIzol reagent (Invitrogen, Carlsbad, CA, USA) according to the manufacturer’s protocol. The first strand of cDNA was synthesized from 0.5 μg of the total RNA by using 100 U of SuperScript™ II RnaseH-Reverse Transcriptase (Invitrogen) and 0.5 μg of dT Primer (Invitrogen). PCR primers with the following sequences were designed by Takara Biotechnology (TaKaRa Biotechnology, Co. Ltd, Dalian, China): rat TLR4 forward primer, 5′-GGCATCATCTTCATTGTCCTTG-3′; rat TLR4 reverse primer, 5′- AGCATTGTCCTCCCACTCG-3′; rat MyD88 forward primer, 5′-AGAGTGGAGAGCAGTGTC-3′; rat MyD88 reverse primer, 5′-GGCAGTAGCAGATGAAGG-3′; rat NF-κB p65 forward primer, 5′-TGCAGGCTCCTGTGCGAGTG-3′; rat NF-κB p65 reverse primer, 5′-TCCGGTGGCGATCGTCTGTGT-3′; rat MCP-1 forward primer, 5′-AGGTCTCTGTCACGCTTCTG-3′; rat MCP-1 reverse primer, 5′-CTGGTGATTCTCTTGTAGTTCTCC-3′. And gene expression was normalized by Glyceraldehyde-3-phosphate dehydrogenase (GAPDH): rat GAPDH forward primer, 5′-ATGGTGAAGGTCGGTGTGAACG-3′; rat GAPDH reverse primer, 5′-CGCTCCTGGAAGATGGTGATGG-3′. PCR reactions containing the fluorescent dye SYBR green were performed in a DNA Engine Opticon RT-qPCR Detection System (Bio-Rad, Hercules, California, USA). The amplification was performed in a volume of 25 μL reaction mixture containing 100 ng cDNA template with the PCR Master Mix reagents kit (Cybrdi, Inc, Rockville, MD, USA). The PCR program involved 45 cycles of 94°C for 10 s, an annealing temperature (from 54°C to 64°C) for 30 s, and 72°C for 30 s. The final results for each sample were normalized relative to the relevant internal control value.

### Western blot analysis

The protein levels of TLR4, MyD88, NF-κB p65 and MCP-1 were detected by Western blotting. The specimens were washed twice in ice-cold PBS and subsequently lysed in RIPA buffer, followed by grinding in liquid nitrogen about 25 ~ 30 min. Supernatant solution was extracted into the centrifuge tube, and insoluble material was removed by microcentrifugation at 12000 r/min for 10 min in 4°C. Protein of lysates were subsequently dissolved in SDS sample buffer (62.5 mM Tris/HCl, pH 6.8, 2% w/v SDS, 10% glycerol, 50 mM dithiothreitol, 0.01% w/v bromophenol blue), electrophoresed on SDS-PAGE with Tris-glycine running buffer, and electrophoretically transferred onto polyvinylidene difluoride membranes using a semi-dry apparatus (Bio-Rad, Hercules, CA, USA). The membranes were blocked with TBS/Tween20 (0.05 m Tris, 0.15 m NaCl, pH 7.6; 1% Tween20) containing 5% w/v non-fat dried milk for 1 h, and then were incubated in TBS/Tween20 with 5% w/v non-fat dried milk supplemented with different rabbit anti-rat monoclonal antibodies (rabbit anti-rat, 1:1000, Santa Cruz Biotechnology, USA) overnight at 4°C. After washed by TBS/Tween20 three times, the membranes were incubated in the dark with the diluted (1:5000) secondary polyclonal antibody (goat anti-rabbit conjugated with peroxidase) in TBS/Tween-20 containing 5% w/v non-fat dried milk at room temperature for 2 h with gentle shaking. Horseradish peroxidase-conjugated anti-β-actin (Santa Cruz Biotechnology, USA) was used as an internal control. Positive antibody interactions were visualized using an ECL-plus kit (Thermo Fisher Scientific Inc, USA) with enhanced chemiluminescence substrate for horseradish peroxidase. The luminescent signals were detected and recorded by a CCD camera in the darkroom, and transmitted to the controller unit. The data was then processed by the analysis equipment linked to the controller unit.

### Statistical analysis

All data were expressed as means ± SD. A Dunnett-t test was used to analyze the significant differences of histopathological staining, TRAP concentration, mRNA expressions and protein levels of the signaling molecules. For multiple comparisons, the LSD *t* test and the Student-Newman-Keuls (SNK) test were used to analyze the statistical differences. A *p*-value less than 0.05 was considered statistically significant.

## Results

### Concentration of TRAP in plasma

**Figure 2:**
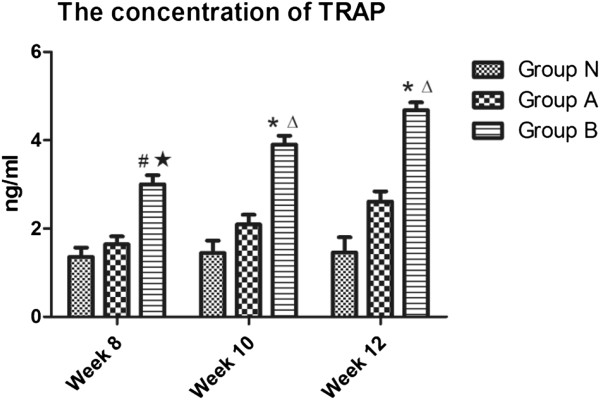
**The concentrations of TRAP by ELISA detection.** In the group **B**, the concentrations of TRAP were mounting steadily during the experimental periods. Compared with the group **N**, the concentrations increased significantly in the group **B**, but no significant in the group **A**. There were significant difference between group **A** and **B**. (P < 0.05: # vs. group **N**; ★ vs. group **A**; P < 0.01: * vs. group **N**; Δvs. group **A**).

Standard curve of TRAP concentration was drawn according to the OD values, and the curve equation was calculated as y = 4.7216x-0.3994 (R^2^ = 0.9975). The actual concentrations were then calculated by the equation and analyzed statistically (Figure 2). It was found that the TRAP concentrations in group B were increasing steadily and had significant difference compared with that in group N during the experimental periods (*p* < 0.01), but the concentrations in group A had little increase and had no obvious significance compared with that in group N (*p* > 0.05). There were significant differences between group A and B (*p* < 0.05). These results suggested that the glucocorticoids could make the osteoclasts activate and the TLR4 antagonists could reduce the activation of osteoclasts.

### Histopathological staining of osteonecrosis

**Figure 3:**
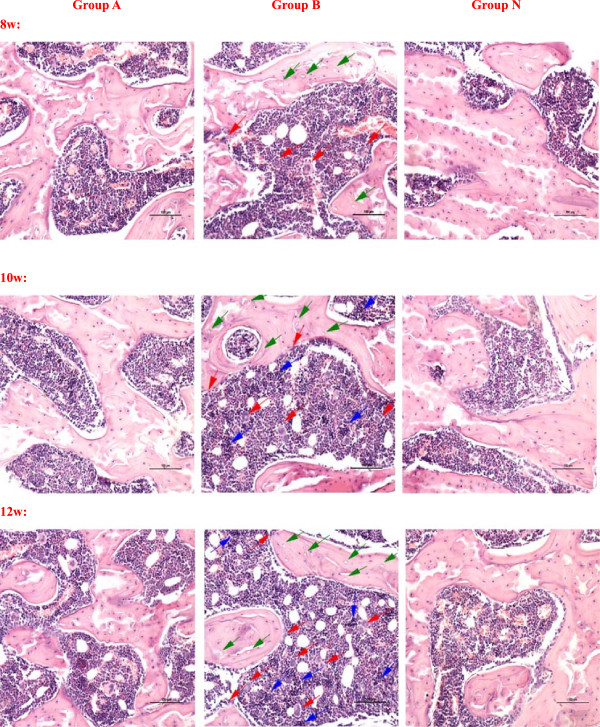
**The observation of femoral head osteonecrosis by Hematoxylin-Eosin staining.** In the group **A**, it showed partial necrotic changes in bone trabeculae and slight accumulation of degenerative or necrotic medullary haematopoietic cells and fat cells in the surrounding bone marrow. A few apparent empty lacunae were observed. The increase of fibroblasts and osteoclasts were not obvious. In the group **B**, the bone trabecular became sparse and fracture, more empty lacunae were seen in it. Haematopoietic cells and fat cells showed necrotic changes, the fibroblasts and osteoclasts increased and accumulated, and many activated osteoclasts were observed and resorbing the necrotic bone trabeculae. The performance of osteonecrosis became more and more serious with time. There was no visible necrosis of bone or bone marrow in the group **N**. (Magnification 200×, scale bar represents 100 μm for all figures, the green arrow stands for the empty lacunae, the red arrow stands for the multinucleated giant cells or osteoclasts, and the blue arrow stands for the necrosis or fibrosis of bone marrow).

Positive diagnosis of osteonecrosis was made on the basis of the diffuse presence of empty lacunae or pyknotic nuclei of osteocytes in the bone trabeculae, accompanied by surrounding bone marrow cell necrosis or myelofibrosis as described in [21]. The mean rates of osteonecrosis in group A and B were 20.8% (5 rats) and 45.8% (11 rats), and 7 rats were in bilateral femoral head necrosis. The mean rate of empty lacunae in group A and B were 10.21% and 36.97%. Figure 3 showed the histopathology of the femoral head of groups A, B and N after haematoxylin-eosin staining. In the group A, it showed partial necrotic changes in bone trabeculae and slight accumulation of degenerative or necrotic medullary haematopoietic cells and fat cells in the surrounding bone marrow. A few apparent empty lacunae were observed. The increase of fibroblasts and osteoclasts were not obvious. In the group B, the bone trabecular became sparse and fracture, more empty lacunae were seen in it. Haematopoietic cells and fat cells showed necrotic changes, the fibroblasts and osteoclasts increased and accumulated, and many activated osteoclasts were observed and resorbing the necrotic bone trabeculae. The performance of osteonecrosis became more and more serious with time. There was no visible necrosis of bone or bone marrow in the group N. These results showed that the model of steroid-induced femoral head osteonecrosis had been successfully established.

### Immunohistochemistry of the signaling molecules

**Figure 4:**
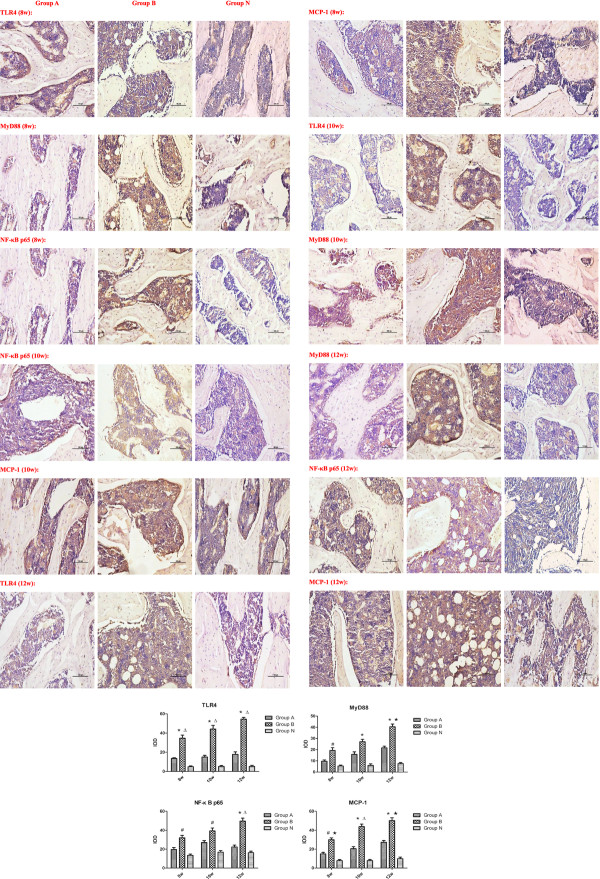
**The positive staining of the signaling molecules in the three groups by immunohistochemistry.** Quantitative analysis demonstrated significant increase in immunolabeling as a percentage of total bone volume for the signaling molecules in the group **B**. Compared with the group **N**, the immunostaining for the signaling molecules in the group **B** increased significantly, but no significant in the group **A**. The expressions of TLR4 and MCP-1 in group **B** had significant differences compared with that in group **A** at each time point. MyD88 and NF-κB p65 expressions in group **B** had significant differences compared with that in group **A** at 12 weeks. (IOD: integrated optical density, P < 0.05: # vs. group **N**; ★ vs. group **A**; P < 0.01: * vs. group **N**; Δ vs. group **A**).

The subchondral portion of the femoral head was the region of interest. Positive immunoreactivity in each group was calculated and compared with the control group. Immunostaining for TLR4, MyD88, NF-κB p65 and MCP-1 increased intensively in group B, but slightly in group A. Compared with the group N, quantitative analysis demonstrated a significant increase in immunolabelling as a percentage of total bone volume for the signaling molecules in group B (*p* < 0.05), but not in group A. (*p*>0.05) (Figure 4). The expressions of TLR4 and MCP-1 in group B had significant differences compared with that in group A at each time point (*p* < 0.05). MyD88 and NF-κB p65 expressions in group B had significant differences compared with that in group A at 12 weeks (p < 0.05). These results showed from both positive and negative aspects that the signaling molecules involved in TLR4 signaling pathway increased and the osteoclasts activated in the femoral head osteonecrosis. TLR4 signaling pathway could play an important role in the pathogenesis of osteonecrosis.

### RT-qPCR of the signaling molecules

**Figure 5:**
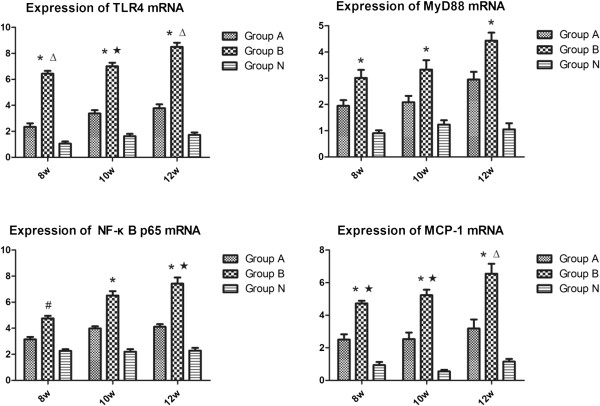
**The mRNA expressions of the signaling molecules in the rats femoral heads of all groups.** The expressions of TLR4, MyD88, NF-κB p65 and MCP-1 increased with time in the group **B**. Compared with the group **N**, there was a significant difference in the group **B**, but no significant in the group **A**. Between groups **A** and **B**, there were significant differences in the mRNA expressions of TLR4 and MCP-1 at each time point and NF-κB p65 at 12 weeks. (P < 0.05: # vs. group **N**; ★ vs. group **A**; P < 0.01: * vs. group **N**; Δ vs. group **A**).

Figure 5 showed the mRNA expressions of the signaling molecules in TLR4 signaling pathway. Compared with the group N, the mRNA expressions of TLR4, MyD88, and NF-κB p65 increased significantly in the group B, but insignificantly in the group A. There was a remarkable difference in the increase of the MCP-1 mRNA expressions between group B and N (*p* < 0.05), and no significant between groups A and N (*p*>0.05). Between groups A and B, there were significant differences in the mRNA expressions of TLR4 and MCP-1 at each time point and NF-κB p65 at 12 weeks (p < 0.05). TLR4 signaling pathway could play an important role in the activation of osteoclast and the pathogenesis of osteonecrosis.

### Protein level of the signaling molecules

**Figure 6:**
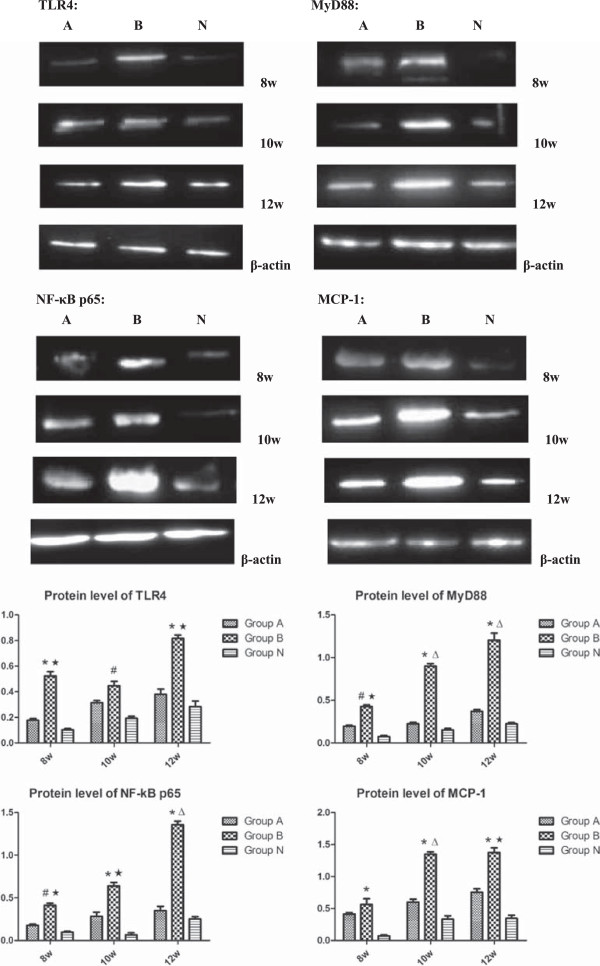
**The protein levels of the signaling molecules in the rats femoral heads of all groups.** Compared with the group **N**, there were remarkable increases in the protein levels of TLR4, MyD88, NF-κB p65 and MCP-1 in the group **B**, and slight increases in the group A. The MCP-1 levels in groups **B** showed most significantly, there was no marked difference between group **A** and group **N**. Between group **A** and group **B**, the protein levels of MyD88 and NF-κB p65 had significant differences at each time point, and TLR4 expression at 8 and 10 weeks, MCP-1 expression at 10 and 12 weeks, all had significant differences. (P < 0.05: # vs. group **N**; ★ vs. group **A**; P < 0.01: * vs. group **N**; Δ vs. group **A**).

Figure 6 presented the increase of protein levels of TLR4, MyD88, NF-κB p65 and MCP-1 in the groups A and B. The protein levels of the signaling molecules in groups B were significantly increased compared with that in group N (*p* < 0.05), whereas, there was no marked difference in the cytokine levels between group A and group N (*p*>0.05). Between group A and group B, the protein levels of MyD88 and NF-κB p65 had significant differences at each time point, and TLR4 expression at 8 and 10 weeks, MCP-1 expression at 10 and 12 weeks, all had significant differences. These results showed from both positive and negative aspects that TLR4 signaling pathway could play an important role in the activation of osteoclast and the pathogenesis of osteonecrosis.

## Discussion

In the present study, we demonstrated that steroid-induced femoral head osteonecrosis was generated in SD rats by administering MP, and the osteoclast activity was more increased in model groups than that in other groups. The signaling molecules involved in TLR4 signaling pathway were expressed highly and significantly in model group compared with that in other groups. Corticosteroids could induced the TLR4 signaling pathway abnormally activated and disturbed the immune response. As a result, the normal balance of bone metabolism was disturbed, a large number of osteoclasts were activated and proliferated, osteonecrosis of femoral head was occurred. On the contrary, the osteonecrosis, osteoclast activity and the signaling molecules involved in TLR4 signaling pathway were showed no significant differences in treatment groups compared with that in control groups.

TLR4, a pattern recognition receptor mainly expressed in monocyte/macrophage and dendritic cells, identifies pathogenic microorganisms, recognizes pathogen associated molecular patterns, and takes part in the innate immunity and adaptive immune response [22,23]. It is also expressed in bone cells, and its activation contributes to TLR ligand-induced osteoclastogenesis and affects osteoclast differentiation [24,25]. It acts as a bridge connecting immune responses and bone metabolism disorders [26]. The signal transduction of TLR4 pathway is MyD88-dependent. MyD88 mediates the activation of NF-κB in the downstream of TLR4 pathway, and NF-κB in turn induces co-stimulatory molecule expression to activate osteoclasts [27]. NF-κB is an important transcriptional signaling molecules regulating a variety of inflammatory and immune genic expressions [28,29]. It has been confirmed that activated NF-κB exists in human endothelial cells, monocytes/macrophages and smooth muscle cells [30]. NF-κB pathway is considered the most significant signaling pathway to transduce the intracellular signal to activate osteoclasts and stimulate osteoclast precursor cells to mature [31]. MCP-1, a cytokine existing in bone marrow, plays an important role in promoting the activation of monocytes/macrophages and causing the cells to gather in the region of bone damage. The activation of monocytes/macrophages can secrete a series of related signaling molecules to mediate osteolysis, which is involved in the bone remodeling process. We utilized the TLR4 antagonist: TAK242 (Resatorvid) as the intervention control group in this study. TAK-242, a cyclohexene derivative, is a novel small-molecule compound that selectively inhibits TLR4 signaling [32,33]. We reported previously that TLR4 is indeed the target protein of TAK-242 and inhibits TLR4 signaling by disrupting the interactions of TLR4 with its adaptor molecules. Although the site to which TAK-242 binds is located in the dimer interface of TLR4, TAK-242 did not affect the dimerization of TLR4 in a protein fragment complementation assay [34]. TAK-242 is a selective inhibitor of signaling from the intracellular domain of TLR4 and represents a novel therapeutic approach to the treatment of TLR4-mediated diseases.

In our study, we used a rat model of steroid-induced femoral head osteonecrosis that had similar histopathological features with the steroid-treated patients to investigate the molecular mechanisms of osteonecrosis. From week 8 to week 12, it was obvious that the histological analysis identified necrotic bone cells surrounded by necrotic bone marrow and necrotic regions with or without being repaired near the epiphyses and subchondral bone in the model groups, but slightly in the treatment groups. However, it should be noted that the structure of the femoral head in rat is different from that in humans. The epiphyseal line of the femur is present during the whole adulthood of rats but not humans. Osteonecrosis of femoral head may easily lead to a collapse of joint in humans but not in rats [35,36]. In our study, the necrosis of epiphysis in the femur was developed in the model groups.

Possibly, steroid-induced osteonecrosis arises due to the activation of the TLR4 signaling pathway that sets the destructive mechanisms in train. Observed in our study, the concentrations of TRAP significantly increased from week 8 to week 12, indicating that the osteoclastogenesis might be activated by corticosteroids. MCP-1 also plays a very important role in activating and aggregating the monocytes/macrophages [37]. Moreover, steroid-induced osteonecrosis arose from the aberrant immune response and the imbalance of bone metabolism. The signaling molecules existed in TLR4 signaling pathway increased significantly with time in the model groups by the corticosteroids administration. These changes might induce an abnormal immunological microenvironment, disturb the balance of immune system and provoke the proliferation of osteoclasts via TLR4 signaling pathway. However, the longest observation time in the present experiment was 12 weeks. A prolonged observation period may better elucidate the pathogenesis of osteonecrosis.

Generally, a rigid skeletal bone is considered as involving a dynamic process of formation and resorption of the bone matrix, carried out by osteoblasts and osteoclasts, respectively. The process is kept in balance to maintain calcium metabolism and skeletal integrity. Osteoclasts can absorb the bone tissues, make the bone resorption exceeds the osteogenesis, destroy the structure of trabecular bone and lead to multiple microfractures. Aaron [38] suggested that the remodeling cycle occured simultaneously when damages appear in the adjacent areas of bone, but the resorption is predominant, causing further destroy of the subchondral bone and ultimately leading to progressive collapse of the trabeculae and finally lead to progressive joint damage [39]. The immune system is spawned in the bone marrow reservoir, and bone also can respond to the certain physiological and immunological demands via releasing cytokines and other signaling molecules into the bloodstream. Therefore, the mechanism of osteonecrosis is possibly associated with immune response, which may involve an abnormality in bidirectional regulation. Excessive corticosteroids administration could disturb the normal immune response and lead to pathological bone resorption. Vink [40] have found that the TLR4 signaling pathway played an important regulated role in the proliferation and differentiation of osteoclasts. Recent studies have elucidated the unanticipated connections between immune system and skeletal system, which have led to the development of a new known field called osteoimmunology, an interdisciplinary research focused on the molecular understanding of the interplay of the skeletal and immune systems [41]. Thus, the immune cells have been implicated in the orthopaedic diseases [42], and the bone remodeling is under the control of several local and systemic factors, including the immune system.

## Conclusions

We have identified that the disorder of immune response via TLR4 signaling pathway plays a role in the pathogenesis of steroid-induced femoral head osteonecrosis. The inhibition of osteoclastogenesis may play a role in reducing the excessive bone loss caused by the disorder of immune response. Therapeutic strategies, such as immunomodulatory biologic drugs specifically targeted at immune cells or signaling molecules, might be a beneficial feature of its clinical use for bone erosion-associated joint diseases.

## Competing interests

The authors declare that they have no competing interests.

## Authors’ contributions

LT made substantial contributions to the conception and design of this manuscript, the acquisition of data, analysis and the interpretation of data and drafting the manuscript. QW carried out the studies of RT-PCR and Western blot, participated in drafting the manuscript. XD carried out the immunohistochemistry and ELISA analysis. WY participated in feeding animals and draw the materials from rats. LF participated in the design of the study and performed the statistical analysis. KW conceived of the study, and participated in its design and coordination, revised the manuscript critically for important intellectual content, and gave the final approval of the version to be published. All authors read and approved the final manuscript.

## Pre-publication history

The pre-publication history for this paper can be accessed here:

http://www.biomedcentral.com/1471-2474/15/18/prepub
